# Supplementary material for: Analysis of Astroglial Secretomic Profile in the Mecp2-Deficient Male Mouse Model of Rett Syndrome
Source: Int J Mol Sci. 2021 Apr 21;22(9):4316. doi: 10.3390/ijms22094316 (PMC8122273; doi:10.3390/ijms22094316)
Supplement: Supplementary file 1 [file ijms-22-04316-s001.zip › ijms-1157316suplementary from proof.pdf]

FIGURE S1

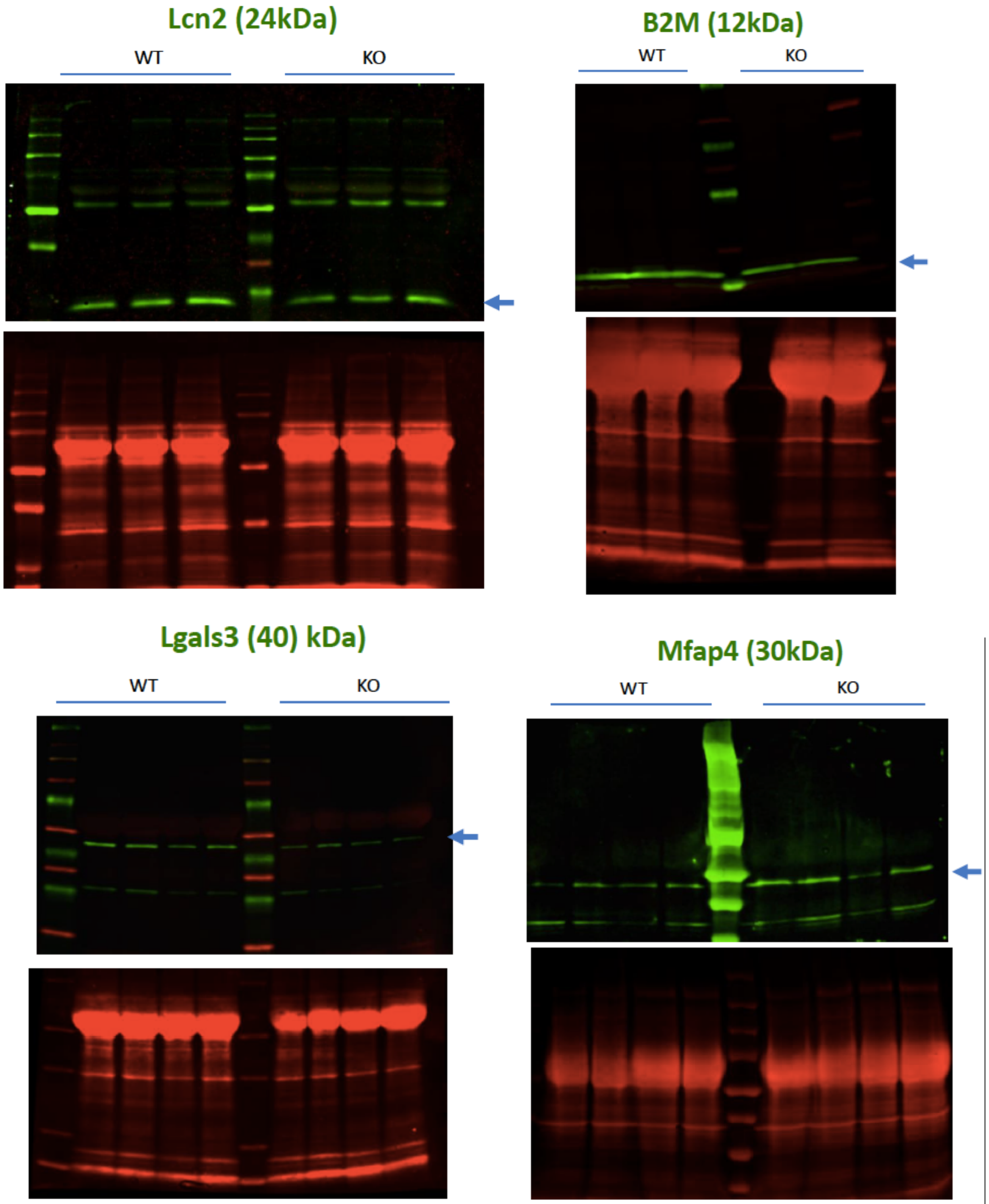
Lcn2 (24kDa)
WT
KO
B2M (12kDa)
WT
KO
Lgals3 (40) kDa)
WT
KO
Mfap4 (30kDa)
WT
KO

FIGURE S2

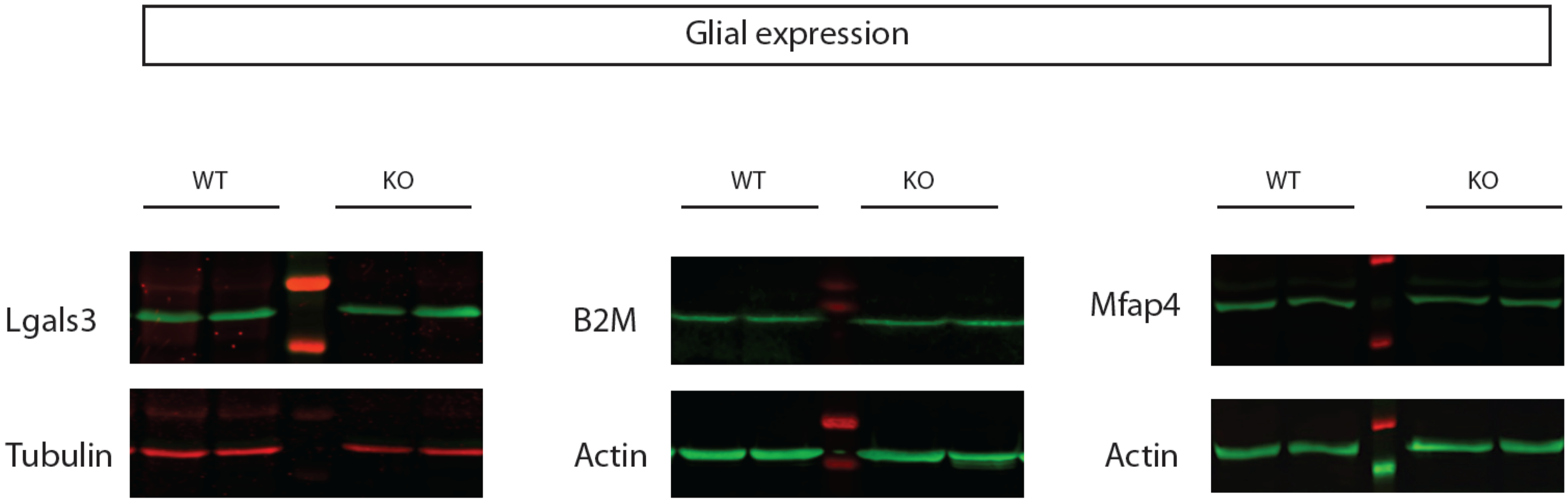
Glial expression
WT
KO
WT
KO
WT
KO
Lgals3
Tubulin
B2M
Actin
Mfap4
Actin

FIGURE S3

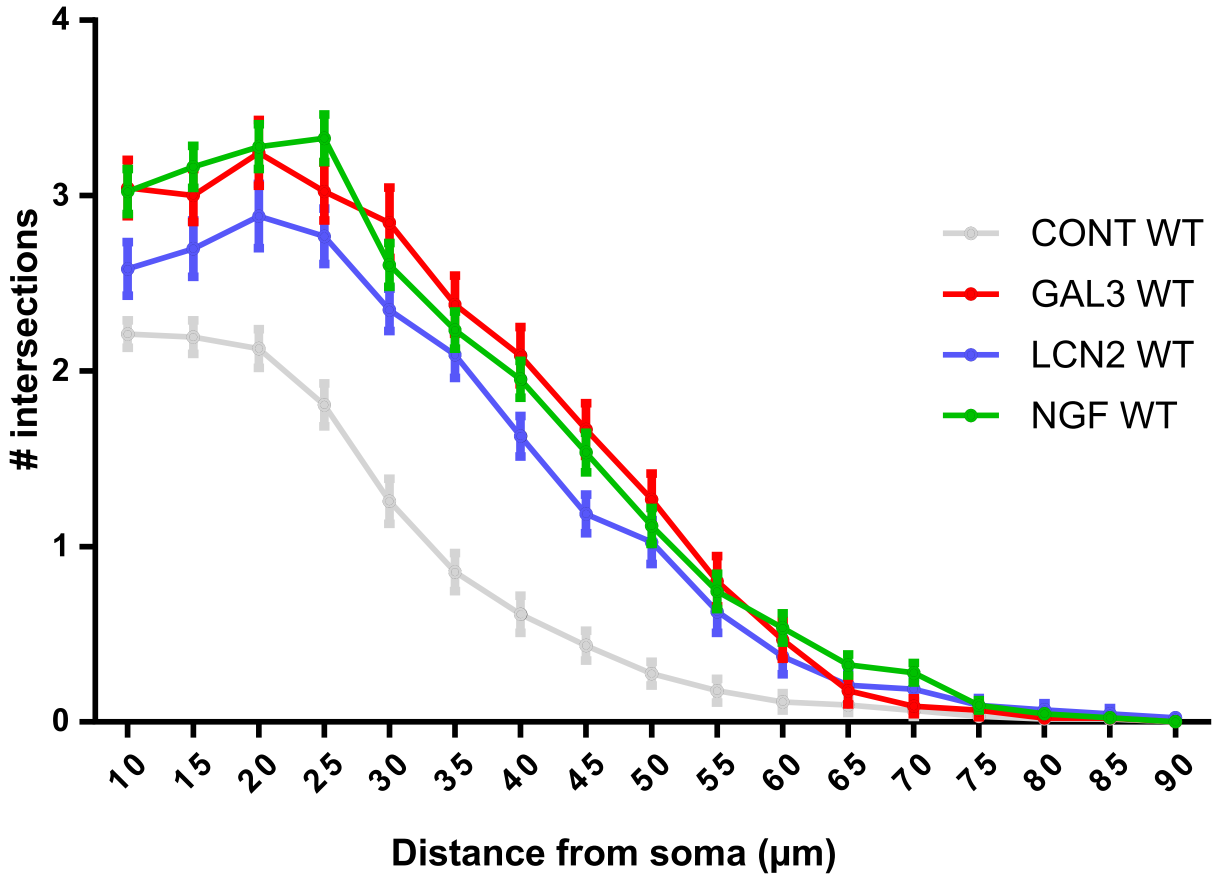

# intersections	10	15	20	25	30	35	40	45	50	55	60	65	70	75	80	85	90
CONT WT vs. LCN2 WT	*	**	***	***	***	***	***	***	***	**	ns	ns	ns	ns	ns	ns	ns
CONT WT vs. GAL3 WT	***	***	***	***	***	***	***	***	***	***	ns	ns	ns	ns	ns	ns	ns
CONT WT vs NGF WT	***	***	***	***	***	***	***	***	***	***	*	ns	ns	ns	ns	ns	ns

FIGURE S4

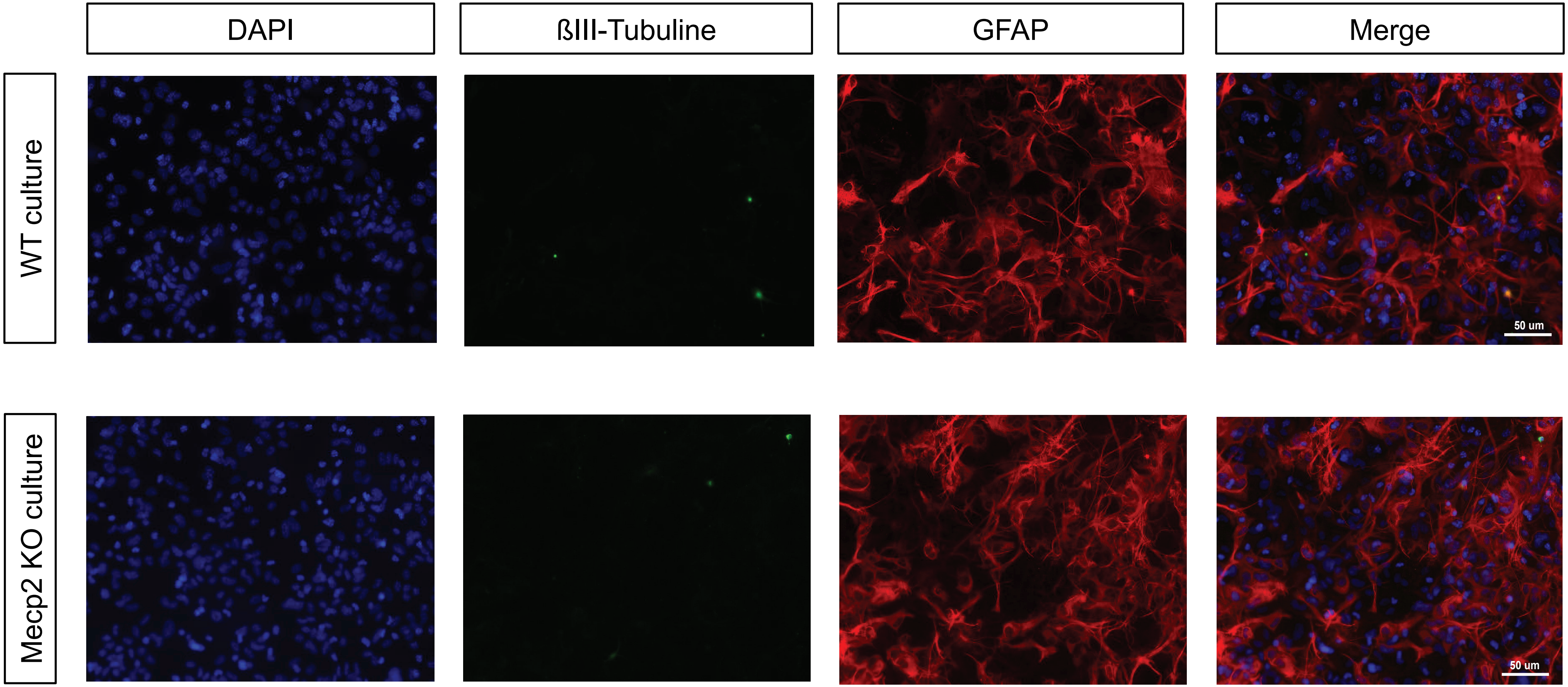
DAPI
ßIII-Tubuline
GFAP
Merge
WT culture
Mecp2 KO culture
50 um
50 um

FIGURE S5

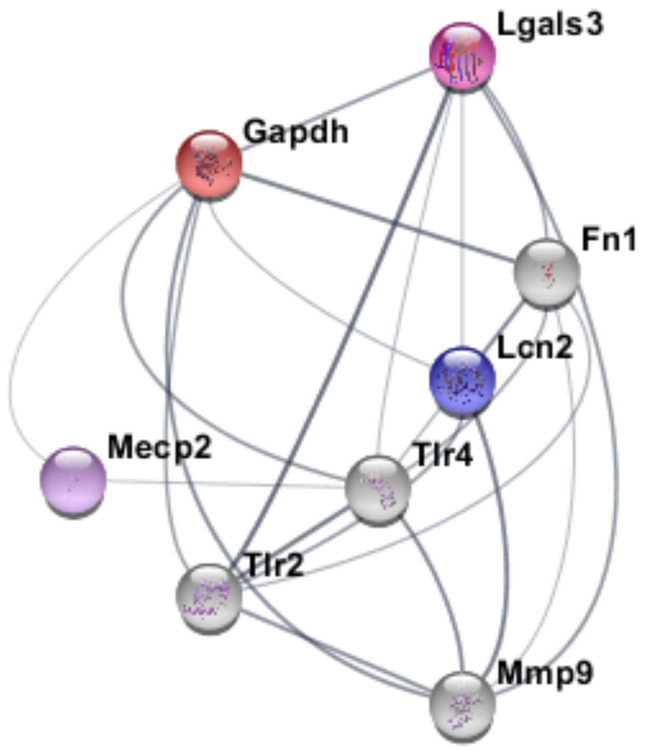

FIGURE S1: Raw data of western blot staining of conditioned-medium from WT and Mecp2 KO astroglial culture for WT and Mecp2 KO samples Lcn2 (n= 3 and 3 respectively), B2M, (n= 3 and 2 respectively), Lgals3 (n= 4 and 4 respectively) and Mfap4 (n= 4 and 4 respectively). Total protein levels obtained with the revert total protein stain method (LI-COR Biotechnology, Bad Homburg, Germany) were used for normalization. Protein specific western blots are shown above their respective total-protein stained membrane.

FIGURE S2: Protein levels of Lgals3, B2M and Mfap4 in WT and Mecp2 KO glial cells. Representative western blot images showing glial expression of Lgals3, B2M and Mfap4. Protein levels were normalized with Tubulin or Actin expression. No difference was found between WT and Mecp2 KO samples (n=3 and n=3 respectively). Lcn2 levels were too low to be analyzed by Western blot test (data not shown).

FIGURE S3: Sholl analysis of WT cortical neurons after specific treatments. Lcn2, Lgals3 and NGF treatments increase number of intersections in Mecp2 KO neurons. Significant differences between conditions and genotypes are summarized in tables. Data are presented as mean ± SEM. Two-way RM Anova followed by Turkey's multiple comparisons test, p<0.05, **p<0.01, **p<0.001, ns: non-significant.
FIGURE S5: Absence of neurons in both WT and Mecp2 KO astroglial culture. Astrocytes are labelled with an antibody anti-GFAP (in red) and neurons are labelled with an antibody anti-BIII tubulin (in green). Nuclei are counterstained by DAPI in blue. Scale bar = 50µm.

FIGURE S4: Absence of neurons in both WT and Mecp2 KO astroglial culture. Astrocytes are labelled with an antibody anti-GFAP (in red) and neurons are labelled with an antibody anti-BIII tubulin (in green). Nuclei are counterstained by DAPI in blue. Scale bar = 50µm.

FIGURE S5: STRING network of secreted Lcn2, Lgals3 and Mecp2 proteins (P16110, P11672 andQ9Z2D6) (Cytoscape v 3.8.0 and StringApp with confidence score cut-off 0.40 and a maximum of 5 additional interactors).

TABLE S1. List of the 557 secreted proteins identified and quantified in glial secretome of mouse Rett syndrome (iTRAQ labelled quantitative proteomic data obtained independently from 2 two-day-old male mice for each condition (Mecp2-KO and wild type))
TABLE S2a. List of the 105 secreted proteins significantly deregulated (DEP) in glial secretome of mouse Rett syndrome (iTRAQ labelled quantitative proteomic data obtained independently from 2 two-day-old male mice for each condition (Mecp2-KO and wild type))

TABLE S2b. Functional annotation of the 105 DEP localization (David Bioinformatics resources 6.8, https://david.ncifcrf.gov/). Gene ontology analysis for cellular compartment.
TABLE S3a. Refined list of 29 secreted proteins significantly deregulated (DEP) in glial secretome of mouse Rett syndrome (iTRAQ labelled quantitative proteomic data obtained independently from 2 two-day-old male mice for each condition (Mecp2-KO and wild type))

TABLE S3b. STRING network analysis of the 29 secreted proteins significantly dysregulated (DEP) in glial secretome of mouse Rett syndrome (Cytoscape v 3.8.0). Raw data used for figure 1

TABLE S4a. Top 6 biological processes obtained from Gene Ontology analysis of the 18 up-regulated proteins (David bioinformatics Resources v6.8)

TABLE S4b. Top 6 biological processes obtained from Gene Ontology analysis of the 11 down-regulated proteins (David bioinformatics Resources v6.8)
